# Music as a Sedative in Neuralgia

**Published:** 1898-09

**Authors:** 


### Music as a Sedative in Neuralgia.

The *British Medical Journal*, in directing renewed attention to the sedative influence of music in neuralgia, states that Mr. Gladstone, during the many weeks of acute neuralgia which ushered in the last phase of his fatal illness, is said to have found great relief in music. Mr. Herbert Spencer is said to have had recourse to music for the relief of nervous disturbance; and the Empress of Austria is reported to have been cured of neuralgia by certain strains of sound repeated at frequent intervals. Many other less illustrious sufferers have had their pain charmed away by the same sweet medicine. The "music cure" had considerable vogue some time ago in Germany, and a special hospital for its systematic application was, we believe, established in Munich.—*Philadelphia Med. Journal.*

